# Predictors of delayed breast reconstruction in the Netherlands: a 5-year follow-up study in stage I–III breast cancer patients

**DOI:** 10.1007/s12282-021-01313-1

**Published:** 2021-11-15

**Authors:** L. S. E. van Egdom, K. M. de Ligt, L. de Munck, L. B. Koppert, M. A. M. Mureau, H. A. Rakhorst, S. Siesling

**Affiliations:** 1 Department of Surgical Oncology, Erasmus MC Cancer Institute, University Medical Center, Rotterdam, The Netherlands; 2 Department of Psychosocial Research, Division of Psychosocial Research and Epidemiology, The Netherlands Cancer Institute, Amsterdam, The Netherlands; 3 Department of Research and Development, Comprehensive Cancer Organisation (IKNL), Utrecht, The Netherlands; 4 Department of Health Technology and Services Research, Technical Medical Centre, University of Twente, Enschede, The Netherlands; 5 Department of Plastic and Reconstructive Surgery, Erasmus MC Cancer Institute, University Medical Center, P.O. 2040, 3000 CA Rotterdam, the Netherlands; 6 Department of Plastic and Reconstructive Surgery, Medisch Spectrum Twente, Enschede, The Netherlands

**Keywords:** Breast cancer, Breast reconstruction, Delayed breast reconstruction, Cancer registry

## Abstract

**Purpose:**

Delayed breast reconstruction (DBR) is a valid option for postmastectomy breast cancer patients who have a desire for breast reconstruction but are not considered suitable for immediate breast reconstruction (IBR). The objective of this study was to investigate the clinical practice and predictors of the use of DBR in the Netherlands.

**Methods:**

Stage I–III breast cancer patients diagnosed from January to March 2012 and treated with mastectomy were selected from the Netherlands Cancer Registry. Routinely collected patient, tumor, treatment and hospital characteristics were complemented with data about DBR up to 2018. Multivariable logistic regression analyses were performed to identify factors independently associated with postmastectomy DBR. Factors associated with time to DBR were identified through Cox regression analyses.

**Results:**

Of all patients who underwent mastectomy (*n* = 1,415), 10.2% underwent DBR. DBR patients more often received autologous reconstruction compared to IBR patients (37.5% vs 6.2%, *p* < 0.001). Age below 50 years (age < 35 OR 15.55, age 35–49 OR 4.18) and neoadjuvant and adjuvant chemotherapy (OR 2.59 and OR 2.83, respectively) were significantly associated with DBR. Mean time to DBR was 2.4 years [range 1–6 years]. Time to DBR was significantly associated with age < 35 years (HR 2.22), and a high hospital volume (HR 1.87).

**Discussion:**

The use of DBR after mastectomy could not be fully explained by age below 50 years, chemotherapy, and hospital volume. Treatment with radiotherapy and adjuvant chemotherapy increased time to DBR. More information about patient preferences is needed to understand the use and timing of reconstruction.

## Introduction

In breast cancer treatment, decisions about surgery are part of a continuum of treatment decisions rather than stand-alone decisions. Although breast-conserving therapy (BCT, breast conserving surgery followed by radiotherapy) has become the central component of surgical breast cancer treatment [1], there is still a considerable proportion of patients who undergo mastectomy [2, 3]. Unfortunately, mastectomy is associated with negative effects on body image and psychosocial well-being [4–6]. Postmastectomy breast reconstruction is considered an important treatment modality in breast cancer care, as it not only restores the breast contour but also provides psychological, psychosocial and functional improvement, including body image and sexuality [5–9]. Patients treated with mastectomy may opt for breast reconstruction, which is either performed during mastectomy as an immediate breast reconstruction (IBR) or as delayed breast reconstruction (DBR) at any given point in time following mastectomy [10].

Multiple factors may affect the timing of breast reconstruction, including both tumor and treatment characteristics as clinical cancer stage, tumor size and localization, comorbidity, smoking, as well as socioeconomic status and individual patient’s and surgeon’s preferences [10–13]. While IBR is not absolutely contraindicated in patients who need to undergo radiotherapy, in the Netherlands DBR is generally preferred for patients with a high risk of postmastectomy radiotherapy, specifically in patients with stage II or III breast cancer [10]. Reasons are that radiotherapy following IBR may not only increase the chance of implant loss, reconstruction failure, or poorer aesthetic outcomes [14, 15], but that IBR may also negatively affect the quality of radiotherapy, specifically if tissue expanders with integrated valves are used [16–18]. Finally, it has been shown that differences exist between Dutch hospitals in organization of breast cancer care, which affects the use of IBR [19].

From a patient perspective, common reasons or preferences for choosing DBR over IBR may range from a preference to focus on oncological treatment first [20, 21] to the unavailability of the desired technique in the facility of breast cancer treatment [22]. Also, patients may feel it is unimportant, unnecessary, nor urgent [20], or they choose to undergo limited surgery as the first procedure [21]. Ultimately, decisions regarding DBR may be relevant from pre-treatment up to years after breast cancer surgery [23].

Trends in IBR have been evaluated extensively, both nationally [24, 25] and internationally [2, 26, 27]. Compared to IBR, where data can be easily studied as IBR is linked to the mastectomy performed, proper collection of DBR-data is more challenging since DBR can be performed years after mastectomy. Consequently, reliable information regarding the current clinical practice of DBR from a national perspective is lacking. Therefore, the objective of the present study was to investigate the clinical practice of the use of DBR in stage I–III breast cancer patients in the Netherlands and the factors predicting its use.

## Methods

### Study population

As DBR may remain relevant years after breast cancer surgery [23], we selected breast cancer patients diagnosed from January to March 2012 from the Netherlands Cancer Registry (NCR) for our nationwide population-based study. Patients with stage I–III disease, treated with a mastectomy, were included. The NCR records data for all newly diagnosed malignancies in the Netherlands since 1989 and incorporates data on patient, tumor and treatment characteristics. Data about breast reconstruction are only routinely collected for IBR; therefore, information regarding DBR was manually and retrospectively retrieved for our cohort. Patients’ electronic health records were checked in 2018, leading to a follow-up period of about five years after diagnosis.

Tumor stage was classified according to the AJCC TNM Classification for Breast Cancer (7th Edition). Topography, morphology, and grade were coded according to the International Classification of Diseases for Oncology, using tumor, node, and metastasis classification system (ICD-O, 3rd edition). Data about recurrent disease was available up to 5 years after diagnosis.

This study was approved by the Privacy Review Board of the NCR.

### Construction of variables

DBR was defined as any reconstruction performed at any other date after mastectomy. To ensure a complete overview, both patients treated with mastectomy only and IBR (defined as any reconstruction on the same date as the mastectomy) were considered as reference group.

Hospitals were grouped according to hospital of oncologic surgery and hospital of reconstruction. The surgical volume of a hospital was defined as the annual number of breast cancer patients in 2012, divided into low-volume < 175 (*n* = 51), mid-range volume 175–245 (*n* = 29), and high-volume > 245 (*n* = 19). Hospitals were categorized as either academic hospitals (including cancer centers, *n* = 8), teaching hospitals (*n* = 44) and general hospitals (*n* = 51). Both academic and teaching hospitals provide medical training to surgical residents. Plastic surgery training is provided in a more limited number of specific hospitals.

### Statistical analyses

Patient, tumor, treatment and hospital characteristics were summarized per treatment group and compared using Pearson Chi-square tests (two-sided). Multivariable logistic regression analyses were used to determine factors that were independently associated with use of breast reconstruction in contrast to mastectomy alone, controlled for patient, tumor, and treatment characteristics. In addition, to determine factors predicting DBR, a Cox regression analysis was performed that took the time between mastectomy and DBR into account and was also controlled for patient, tumor, and treatment characteristics. Included variables which were selected based on literature [10, 11] were: age at time of surgery, clinical tumor and nodal stage, morphology, differentiation grade, chemotherapy, radiotherapy, hospital type, and hospital volume. Conditions of proportionality were analyzed graphically. In multivariable analyses, P-values < 0.05 were considered as statistically significant. All analysis was performed using STATA (version 14) [28].

### Sample size

To enable regression analyses, Harris’ rule of thumb (1985) prescribes a minimum of ten participants per predictor variable in equations including six or more variables [29]. We expected to include 10–15 independent variables in our multivariable regression, requiring a minimum of 150 DBR patients. In a similar cohort study performed in Denmark, that has an identical nationwide cancer registry, 10.1% of women received DBR in the years following diagnosis (1999–2006, follow-up to 2009) [30]. By including 1500 patients treated with mastectomy, about a quarter of annually newly diagnosed breast cancer patients treated with mastectomy in the Netherlands, we expected to include enough DBR patients. In 2012, quarterly rates of mastectomy and IBR were constant, suggesting generalizability of DBR-rates over a similar period.

## Results

### Patient characteristics

Of all patients diagnosed with stage I–III breast cancer between January and March 2012, 36% of patients (*n* = 1,415) had been surgically treated with mastectomy (**Table ****1**). Of these patients, 144 (10.2%) patients received DBR.

**Table 1:** Patient, tumor, treatment, and hospital characteristics for patients treated with mastectomy (n = 1,415), diagnosed between January and March 2012 (n,%)

		DBR (*n* = 144)	%	IBR (*n* = 194)	%	MAS (*n* = 1077)	%	p*
*Patient characteristics*								
Age in years (at diagnosis)	< 35	22	15.3%	19	9.8%	18	1.7%	** < 0.001**
	35–49	73	50.7%	73	37.6%	169	15.7%	
	50–75	49	34.0%	99	51.0%	609	56.5%	
	75 +	0	0.0%	3	1.5%	281	26.1%	
	Mean (range)	47.4	25.1–74.9	51.1	26.7–78.8	64.5	26.3–96.5	n/a
Tumor characteristics								
Stage (clinical)	I	43	29.9%	93	47.9%	336	31.2%	** < 0.001**
	II	78	54.2%	81	41.8%	585	54.3%	
	III	17	11.8%	5	2.6%	112	10.4%	
	Unknown	6	4.2%	15	7.7%	44	4.1%	
Clinical tumor size (cT)	0/IS	0	0.0%	2	1.0%	2	0.2%	** < 0.001**
	cT1	58	40.3%	99	51.0%	401	37.2%	
	cT2	58	40.3%	69	35.6%	473	43.9%	
	cT3	19	13.2%	10	5.2%	98	9.1%	
	cT4	2	1.4%	0	0.0%	55	5.1%	
	Missing	7	4.9%	14	7.2%	48	4.5%	
Grade	Grade I	15	10.4%	38	19.6%	145	13.5%	0.090
	Grade II	60	41.7%	79	40.7%	446	41.4%	
	Grade III	48	33.3%	46	23.7%	345	32.0%	
	Unknown	21	14.6%	31	16.0%	141	13.1%	
HER2 status	Positive	29	20.6%	33	18.1%	171	16.1%	0.393
	Negative	112	79.4%	146	80.2%	877	82.4%	
	Unclear	0	0.0%	3	1.6%	16	1.5%	
Hormone receptor status	Positive	99	68.8%	127	68.6%	654	61.1%	0.053
	Mixed	15	10.4%	28	15.1%	201	18.8%	
	Negative	30	20.8%	30	16.2%	215	20.1%	
ER status^a^	Negative	31	21.5%	31	16.8%	221	20.7%	0.438
	Positive	113	78.5%	154	83.2%	849	79.3%	
PR status^a^	Negative	44	30.6%	57	30.8%	410	38.3%	**0.043**
	Positive	100	69.4%	128	69.2%	660	61.7%	
Multifocality	No	91	63.2%	129	69.7%	745	69.6%	0.447
	Yes	52	36.1%	56	30.3%	318	29.7%	
Lymph node status	N0	61	42.4%	132	68.0%	491	45.6%	** < 0.001**
	N +	83	57.6%	58	29.9%	552	51.3%	
	Missing	0	0.0%	4	2.1%	34	3.2%	
Follow-up characteristics								
Mean time to recurrence (in days)	Mean (SD)	1070	428	1012	466	884	447	n/a
Type of recurrence	Local	4	2.7	4	2.2	40	1.5	n/a
	Regional	4	2.7	3	1.6	57	2.1	
	Metastasis	10	6.8	7	3.8	196	7.1	
Treatment characteristics								
Chemotherapy	Yes	120	83.3%	120	61.9%	522	48.5%	** < 0.001**
	Neoadjuvant	94	78.3%	89	74.2%	390	74.7%	0.758
	Adjuvant	23	19.2%	27	22.5%	122	23.4%	
	Both	3	2.5%	4	3.3%	10	1.9%	
Endocrine therapy	Yes	102	70.8%	121	62.4%	707	65.6%	0.267
Radiotherapy	Yes	52	36.1%	28	14.5%	359	33.3%	** < 0.001**
	Before BR	48	92.0%	0	0.0%	n/a	n/a	** < 0.001**
	After BR	4	8.0%	28	100.0%	n/a	n/a	
Hospital characteristics								
Hospital type (hospital of oncologic treatment)^b^	General hospital	60	41.7%	56	28.9%	385	35.7%	** < 0.001**
	Teaching hospital	76	52.8%	111	57.2%	631	58.6%	
	Academic hospital	8	5.6%	27	13.9%	61	5.7%	
Hospital type (hospital of breast reconstruction)^b^	General hospital	43	30.4%	55	28.4%	n/a	n/a	0.796
	Teaching hospital	81	58.0%	112	57.7%	n/a	n/a	
	Academic hospital	16	11.6%	27	13.9%	n/a	n/a	
Hospital volume (hospital of oncologic treatment) ^c^	Low	50	34.7%	48	24.7%	380	35.3%	**0.006**
	Middle	46	31.9%	60	30.9%	361	33.5%	
	High	48	33.3%	86	44.3%	336	31.2%	

DBR, mastectomy with delayed breast reconstruction; IBR, mastectomy with immediate breast reconstruction; MAS, mastectomy alone

^*^Chi-square tested

^a^ Only available if hormone receptor status was tested

^b^ Hospitals were categorized as either general, teaching, or academic hospitals. Cancer-specialized centers were included in the category of academic hospitals

^c^ Number of surgical treated non-metastatic breast cancer patients in 2012, categorized as low (≤ 175), medium (175 < 245), and high (> = 245) volume

Breast cancer patients with DBR significantly differed from patients with IBR or mastectomy alone. DBR patients had a significantly lower mean age (p < 0.001) and were significantly more often diagnosed with a higher clinical stage (stage III: 11.8%; T2-stage: 40.3% or T3-stage: 13.2%) and nodal involvement (57.6%; p < 0.05). Statistically significant differences were found between all groups for treatment characteristics, including radiotherapy and chemotherapy (*p* < 0.001), as well as for hospital type (*p* < 0.001*)* and hospital volume (*p* < 0.006, **Table ****1**).

**Fig. 1:**
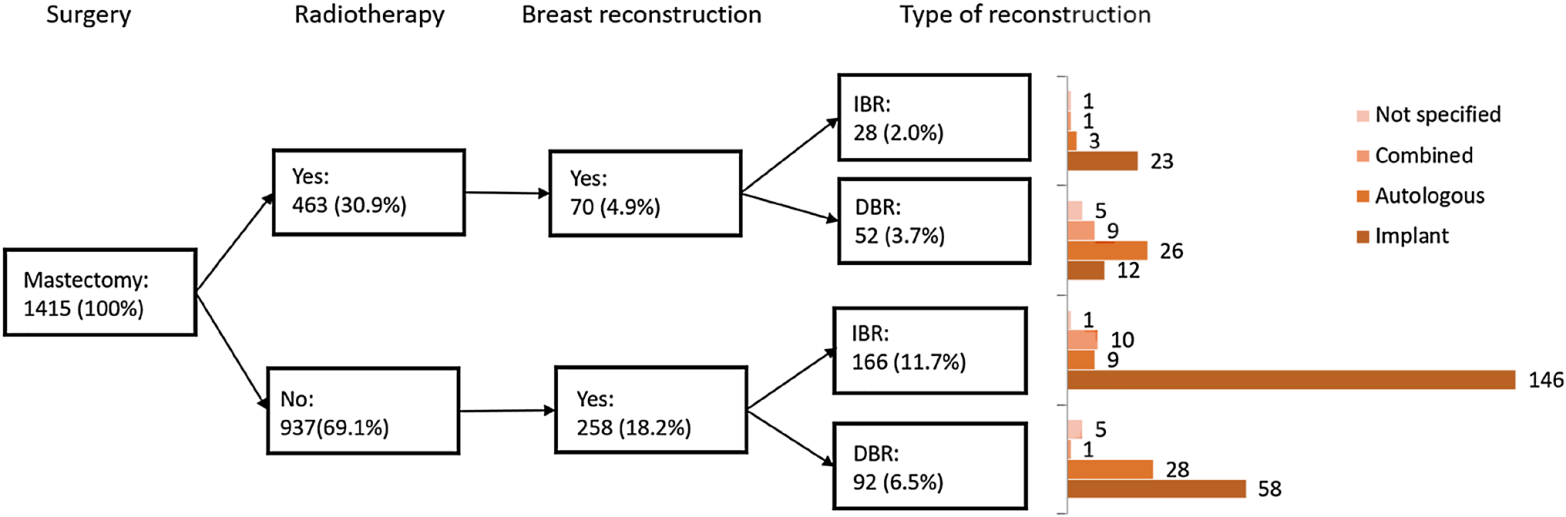
Treatment characteristics for surgical treated patients diagnosed between January and March 2012. IBR, mastectomy with immediate breast reconstruction; DBR, mastectomy with delayed breast reconstruction. Percentages compared to population of patients treated with mastectomy (*n* = 1415). Absolute numbers of type of reconstruction are reported at the end of the corresponding bar

Figure. 1 shows a flowchart of the treatment characteristics for all breast reconstruction patients following mastectomy. Most patients who had undergone breast reconstruction (either IBR or DBR) were not treated with adjuvant radiotherapy.

### Factors associated with the use of DBR

DBR patients significantly more often received mastectomy at general hospitals compared to IBR patients (41.7% versus 28.9%, respectively, *p* < 0.001). Implant-based DBR was performed most frequently (*n* = 70, 48.6%), followed by autologous DBR (*n* = 54, 37.5%; **Table ****2**). However, autologous reconstructions were performed significantly more often in DBR patients than in IBR patients (6.2%), where implant-based reconstructions were leading (82.1%).

**Table 2:** Type of breast reconstruction performed (n,%) for patients diagnosed between January and March 2012

	DBR (*n* = 144)	%	IBR (*n* = 194)	%	p*
Autologous	54	37.5%	12	6.2%	** < 0.001**^**b**^
Combined autologous and implants	10	6.9%	11	5.7%	
Implants	70	48.6%	169	82.1%	
Not specified	10	6.9%	2	1.0%	

DBR, mastectomy with delayed breast reconstruction; IBR, mastectomy with immediate breast reconstruction

^*^Chi-square tested

A multivariable logistic regression analysis for factors relate to any breast reconstruction (including both DBR and IBR) in contrast to mastectomy alone was performed. The use of BR was significantly and positively associated with age < 35 years (OR 14.74, CI 7.42 – 29.26, *p* < 0.001), age 35–49 (OR 4.33, CI 3.18–5.89, *p* < 0.001), receiving chemotherapy (either neoadjuvant (OR 2.25, CI 1.21–4.20, p = 0.011) or adjuvant (OR 1.62, CI 1.12–2.36, *p* = 0.011)), and treatment in an academic hospital (OR 1.85, CI 1.11—3.09, *p* = 0.018) or in a hospital of higher volume (middle volume: OR 1.64, CI 1.12 – 2.42, *p* = 0.010; high volume: OR 2.42, CI 1.59 – 3.69, *p* < 0.001). Significantly negatively associated with the use of BR were age 75 + (OR 0.06, CI 0.02 – 0.20, *p* < 0.001), positive lymph node status (OR 0.54, CI 0.36 – 0.79, *p* = 0.002) or unknown lymph node status (OR 0.17, CI 0.04 – 0.69, *p* = 0.014), radiotherapy (OR 0.03, CI 0.02 – 0.04, *p* < 0.001), and treatment in a teaching hospital (OR 0.68, CI 0.47 – 0.99, *p* = 0.046) (**Table ****3**).

**Table 3:** Multivariable logistic regression for the odds of immediate or delayed breast reconstruction (n = 338) versus mastectomy alone (n = 1,077)

		Total *N* = 1.077	BR *N* = 338	Multivariable		p*
				OR	95% CI	
Patient characteristics						
Age	< 35	46	30	14.74	7.42 – 29.26	** < 0.001**
	35–49	323	155	4.33	3.18 – 5.89	** < 0.001**
	50–75	757	150	Ref		
	75 +	289	3	0.06	0.02 – 0.20	** < 0.001**
Tumor characteristics						
Clinical tumor-stage	cT0	4	2	8.78	0.55 – 140.64	0.125
	cT1	558	157	Ref		
	cT2	600	127	0.91	0.67 – 1.24	0.540
	cT3	127	29	1.41	0.79 – 2.53	0.250
	cT4	57	2	0.24	0.05 – 1.24	0.089
	Missing	69	21	1.64	0.91 – 2.99	0.103
Grade	Grade I	198	53	ref		
	Grade II	585	139	0.98	0.66 – 1.46	0.930
	Grade III	439	94	0.77	0.49 – 1.20	0.246
	Unknown	193	52	0.98	0.53 – 1.79	0.938
Lymph node status	N0	684	193	ref		
	N +	693	141	0.54	0.36 – 0.79	**0.002**
	Not assessed/unknown	38	4	0.17	0.04 – 0.69	**0.014**
Treatment characteristics						
Chemotherapy	No	653	98	Ref		
	Yes, adjuvant	573	183	1.62	1.12 – 2.36	**0.011**
	Yes, neoadjuvant	172	50	2.25	1.21 – 4.20	**0.011**
Radiotherapy	No	976	258	ref		
	Yes	439	80	0.03	0.02 – 0.04	** < 0.001**
Endocrine therapy	No	485	115	Ref		
	Yes	930	223	1.02	0.75 – 1.40	0.892
Hospital factors						
Hospital type oncologic treatment^a^	General hospital	501	116	ref		
	Teaching hospital	818	187	0.68	0.47 – 0.99	**0.046**
	Academic hospital	96	35	1.85	1.11 – 3.09	**0.018**
Hospital volume^b^	Low	478	98	ref		
	Middle	467	106	1.64	1.12 – 2.42	**0.010**
	high	470	134	2.42	1.59 – 3.69	** < 0.001**
Goodness-of-fit	Prob > chi2 = 0.98					
Area under ROC curve	0.89					

BR, mastectomy with breast reconstruction (both immediate and delayed); cT, clinical tumor-stage; MDT, multidisciplinary team meeting

^*^Chi-square tested

a Hospitals were categorized as either general, teaching, or academic hospitals. Cancer-specialized centers were included in the category of academic hospitals

b Number of surgical treated non-metastatic breast cancer patients in 2012, categorized as low (≤ 175), medium (175–245), or high (> 245) volume

As part of a sensitivity analysis, a multivariable logistic regression analysis was performed to determine factors that were independently associated with use of DBR in contrast to mastectomy alone. For DBR specifically, only age < 35 years (OR 15.55, CI 8.37–28.93, *p* < 0.001), age 35–49 (OR 4.18, CI 2.84–6.17, *p* < 0.001), and receiving neoadjuvant chemotherapy (OR 2.59, CI 1.39–4.84, *p* < 0.001) or adjuvant chemotherapy (OR 2.83, CI 1.75–4.56, *p* < 0.001) were significantly and positively associated (data not shown).”

### Factors associated with the time between mastectomy and DBR

**Fig. 2:**
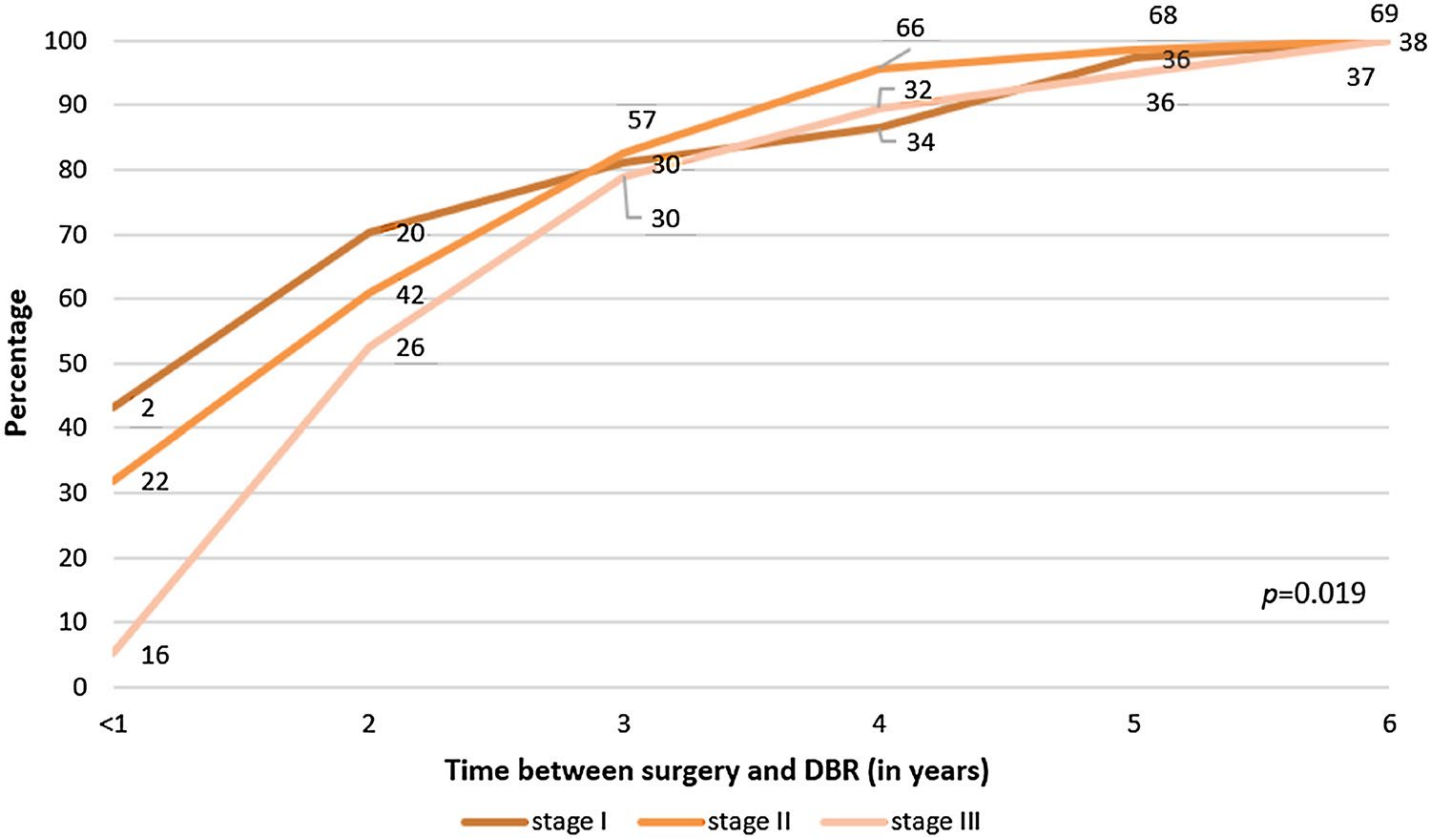
Time (in years) between mastectomy and DBR per TNM-stage; cumulative number of DBR (*n* = 144). DBR, mastectomy with delayed breast reconstruction. Absolute cumulative numbers of patients with DBR are reported per year over each line

Mean time to DBR was 710 days (**Table ****1**). In Fig. 2, time to DBR was categorized by TNM-staging. With an increasing tumor stage, DBR was performed later in time, starting ≥ 1 year following mastectomy (*p* = 0.019). Patients treated with radiotherapy received DBR approximately 1 year later than patients without radiotherapy, with a mean time between diagnosis and DBR of 2.9 years (SD 1.26) versus 2.1 years (SD 1.23), respectively (*p* = 0.002; results not shown in Fig. 2).

In the multivariable Cox regression analyses, DBR was significantly associated with age < 35 years (HR 2.22, CI 1.12–4.39, p = 0.024), and a high hospital volume (HR 1.87, CI 1.07–3.26, p = 0.028) (**Table ****4**).

**Table 4:** Cox regression for the time between mastectomy and delayed breast reconstruction

		*N* = 144	Multivariable		p*
			HR	95% CI	
Patient characteristics					
Age	< 35	17	2.22	1.12 – 4.39	**0.024**
	35–49	77	1.42	0.90 – 2.24	0.136
	50–75	50	ref		
	75 +	0		omitted	
Tumor characteristics					
Clinical tumor-stage	cT0	0	–	–	–
	cT1	58	Ref		
	cT2	58	0.79	0.51 – 1.22	0.289
	cT3	19	1.07	0.53 – 2.13	0.856
	cT4	2	1.50	0.30 – 7.55	0.625
	missing	7	1.65	0.70 – 3.91	0.254
Lymph node status	N0	61	Ref		
	N +	83	0.86	0.53 – 1.37	0.519
Grade	Grade I	15	ref		
	Grade II	60	0.73	0.38 – 1.38	0.331
	Grade III	48	0.90	0.45 – 1.79	0.770
	Unknown	21	0.78	0.25 – 2.42	0.673
Treatment characteristics					
Chemotherapy	No	24	Ref		
	Yes, adjuvant	94	0.54	0.29 – 1.01	0.052
	Yes, neoadjuvant	23	0.89	0.34 – 2.38	0.822
Radiotherapy	No	92	Ref		
	Yes	52	0.64	0.39 – 1.05	0.080
Endocrine therapy	No	42	Ref		
	Yes	102	0.84	0.54 – 1.32	0.449
Hospital factors					
Hospital type (hospital of oncologic treatment)^a^	General hospital	60	ref		
	Teaching hospital	76	0.66	0.37 – 1.20	0.172
	Academic hospital	8	0.53	0.15 – 1.89	0.329
Hospital type (hospital of DBR)^a^	General hospital	43	ref		
	Teaching hospital	81	0.85	0.52 – 1.40	0.520
	Academic hospital	16	1.16	0.48 – 2.78	0.739
Hospital volume (hospital of oncologic treatment)^b^	Low	50	ref		
	Middle	46	1.02	0.63 – 1.67	0.929
	High	48	1.87	1.07 – 3.26	**0.028**

Prob > chi 2 = 0.046

cT, clinical tumor-stage

^*^Chi-square tested

^a^ Hospitals were categorized as either general, teaching, or academic hospitals. Cancer-specialized centers were included in the category of academic hospitals

^b^ Number of surgical treated non-metastatic breast cancer patients in 2012, categorized as low (≤ 175), medium (175–245), or high (> 245) volume

## Discussion

The objective of the current study was to investigate the breast cancer patient population opting for DBR in the Netherlands. Based on one-quarter of newly diagnosed patients treated with mastectomy in 2012, DBR was performed in 10.2% (144/1,415) of patients, which is consistent with DBR rates of 9.3–13% from European literature (Denmark, the UK; 1999–2009) [30, 31]. Breast cancer patients with DBR significantly differed from patients with IBR or mastectomy alone. DBR patients were significantly younger, were more often diagnosed with stage II breast cancer and axillary lymph node metastases and were more often treated with chemotherapy or radiotherapy. This corresponds with the rationale that patients who are considered eligible for IBR are generally diagnosed with stage I breast cancer, with a good prognosis and a negative sentinel lymph node without the indication for axillary lymphadenectomy or radiotherapy [32]. In addition, the minority of DBR and mastectomy alone patients were treated at an academic hospital, unlike IBR in which the majority was treated in teaching hospitals or academic hospitals. This corroborates with available literature, demonstrating that (immediate) breast reconstruction rates were most probably higher in breast cancer specialized centers and hospitals with a high clinical breast surgery volume, because of high referrals to plastic surgeons [19, 24, 33].

Although not exclusively explaining the use of DBR, age below 50 years and treatment with (neoadjuvant) chemotherapy were significantly associated with DBR. This is in contrast to current literature. Initiation of adjuvant chemotherapy is recommended within 6–12 weeks after mastectomy [10, 34–36], and a recent large Dutch population-based study found that IBR did not reduce the likelihood of receiving adjuvant chemotherapy within 9 or 12 weeks following mastectomy [37]. This suggests that IBR does not delay the initiation of adjuvant chemotherapy to a clinically relevant extent [37], and thus DBR is not per se preferred over IBR if chemotherapy is indicated. Furthermore, literature has shown younger age is mainly related to higher IBR-rates [24, 38, 39], not to higher DBR-rates, and IBR-rates decrease significantly with increasing age [40]. Therefore, both associations may seem unexpected. However, our results could be explained by the following:

One could argue that the decision for DBR is not completely isolated. First, the decisions for IBR and DBR are interdependent, or as mentioned in our introduction section, part of a continuum of decisions. There are several reasons to prefer IBR over DBR, including cosmetic result and organizational benefits [8, 10, 23]. Patients who prefer a reconstruction but have a contra-indication for IBR, will most likely opt for DBR as a second best solution. However, risk-factors for surgical and medical complications as smoking, BMI > 30 kg/m2, older age and co-morbidities (i.e. diabetes mellitus, hypertension), can negatively affect the outcomes of both IBR and DBR [10, 13]. This is different for radiotherapy, which is considered as a relative contraindication for IBR in particular. The Dutch breast reconstruction guideline states it is preferable not to perform IBR with an implant if there is a high likelihood of postmastectomy radiotherapy [10]. The decision for DBR is, therefore, conditional to the decision regarding IBR, which may explain why we found no significant association of radiotherapy with DBR. This is illustrated by our sensitivity analysis. Radiotherapy was strongly and negatively associated with receiving any breast reconstruction (IBR or DBR) in contrast to mastectomy alone (**Table ****3**). Radiotherapy has been reported as the most common reason to delay breast reconstruction until the acute side-effects of radiotherapy have been resolved, preferring DBR over IBR [10, 16, 18, 32]. Our Cox regression analysis demonstrated that the time between mastectomy and DBR was longer when radiotherapy was given (although just not significant); most patients were scheduled for DBR at least 2 years after radiotherapy completion.

The same may apply for age. Of all patients with a contra-indication for IBR, young patients may still opt for DBR, whereas older patients may be satisfied with mastectomy alone. This is partly explained by both patients’ preferences and clinicians’ beliefs. As older patients are more likely to have significant comorbidities, clinicians may find younger patients more eligible to undergo breast reconstruction [13, 40]. Moreover, one may speculate that older patients more easily accept the loss of their breast(s) or may not want to undergo major surgery. Younger patients in contrast may be more aware of breast reconstruction possibilities [24] and may be more assertive to discuss this option with their physician [23].

Second, treatment practice has changed since 2012. In the past, (neo)adjuvant therapies were considered contra-indications for IBR. Whether a reconstruction should be delayed if a patient requires radiotherapy remains a matter of debate. Chemotherapy, on the other hand, is no longer considered a major contraindication for IBR [41]. Results of a recent systematic review showed that neoadjuvant chemotherapy was not found to affect surgical complications as seroma, wound complications, skin or nipple necrosis, flap ischemia or loss, and implant loss [42]. In our cohort, DBR patients were more often treated with chemotherapy. Chemotherapy may be a proxy for disease severity and the prognosis of recurrence risk: DBR patients significantly more often had stage III disease and larger pre-treatment tumors. Therefore, reluctance towards IBR may have caused physicians to advice DBR instead. At that time, IBR was more cautiously offered to patients indicated for chemotherapy [43], while current evidence-based guidelines state chemotherapy is not a contra-indication for IBR [37, 41]. In our study we revealed a longer time between DBR in case of chemotherapy. The reason for this is not clear but could be patients were initially reluctant to undergo yet another stressful, and often mayor, surgical treatment, while still recovering from various significant physical complaints.

Implant-based breast reconstruction was the most frequently used technique (48.6% DBR; 82.1% IBR) in our cohort. Nowadays, however, autologous reconstructions are increasingly recommended [10], as lower rates of total reconstruction failure and better long-term patient satisfaction with aesthetic outcome compared to implant reconstruction have been reported [6, 44, 45]. In our radiated sub-population, the majority had received autologous breast reconstruction. The Dutch evidence-based guideline for breast reconstruction states that for DBR after radiotherapy it is preferable not to perform reconstruction with an implant only due to the high risk of implant loss [10], but rather add non-irradiated tissue to cover the implant or perform a full autologous reconstruction.

The nationwide and population-based character is a major strength of our study. However, the data obtained for this study were restricted to the time period from January to March 2012. Although this limits the size of our sample, our sample size calculation substantiates the number of included patients in our cohort. Because DBR is not routinely registered in the NCR, data had to be manually collected retrospectively over a time period of 5 years of follow-up. Still, within this quarter, patients who received DBR in a hospital different from were mastectomy was performed, may have been lost to follow-up. Patients may have decided independently for DBR at another hospital, leaving no paper trail at the hospital where the mastectomy was performed. These referral patterns are not easily identified by NCR’s registrars, especially when time since diagnosis passes, probably resulting in a somewhat smaller number of identified DBR patients. However, in a study on IBR a 5% hospital transfer was seen [46], which implies little incompleteness in our study. Active follow-up for all patients in the NCR is advisable.

Several latent variables may have accounted for the reduced explanatory power of our multivariable logistic regression model for the use of DBR. Factors as patients’ preferences and psychological issues, behavior (smoking, BMI), comorbidities (morbidity obesity, diabetes mellitus), socioeconomic status, surgeons’ beliefs and/or hospital organizational factors probably also affect the use of DBR, as they do in IBR [12, 19, 24], but are not collected in detail in the NCR. The lack of this information may have weakened our results and should be explored in future studies. For IBR patients, multiple hospital organizational factors were identified that could possibly also affect the use of DBR after mastectomy for stage I–III breast cancer in the Netherlands, including hospital type and volume, employment of a plastic surgeon, referral to a plastic surgeon, and the structural attendance of a plastic surgeon at the MDT [19].

The present study provides an overview of the use of DBR within a Dutch population of breast cancer patients treated with mastectomy in 2012, followed up till 5 years after diagnosis. Studies with long-term events since cancer treatment as primary outcome, such as recurrent disease, face the fact that clinical practice usually has changed over time since diagnosis because of improvements in cancer treatments. Similarly, this should be kept in mind when interpreting conclusions about DBR. Currently, BCT is considered at least equally safe as mastectomy [1], resulting in an increasing number of breast cancer patients treated with BCT [47]. However, the absolute number of DBR has also risen to 2,300 per year in 2019 (opendisdata, NABON Breast Cancer Audit). In addition, IBR-rates in mastectomy patients have increased over the past years as well. In 2019, 12,852 women were diagnosed with invasive breast cancer, of whom 4,252 patients were treated with a mastectomy [3]. Twenty-eight percent of these women received IBR, compared to only 16% in 2012 (opendisdata, NABON Breast Cancer Audit). Regarding which type of breast reconstruction is used, recent concerns about the association between the use of breast implants and anaplastic large cell lymphoma (ALCL) [48] and its relatively high prevalence of 4.1 reported cases per million inhabitants in the Netherlands [49] could potentially further shift the preference to autologous reconstructions.

A population-based overview was given of mastectomy patients opting for DBR. Our study is a starting point for future practice evaluation. In order to answer aforementioned questions, data on DBR should be registered on regular basis similar to IBR, taking into account the fact that DBR can be performed until years after the mastectomy. Future research is needed to identify the trend of DBR within the Netherlands over the past years, the variation between hospitals in performing DBR after mastectomy, and the effects of patients’ and surgeons’ preferences, taking behavioral factors, comorbidities and socioeconomic status into account.

## Abbreviations

DBR: Delayed breast reconstruction
IBR: Immediate breast reconstruction
BCT: Breast conserving therapy
NCR: Netherlands Cancer Registry
MDT: Multidisciplinary team meeting
